# Transcriptional Responses to Fire in Three Savanna Grass Species: Candidate Pathways for Fire-Stimulated Flowering

**DOI:** 10.3390/ijms27219590

**Published:** 2026-10-27

**Authors:** Utsab Ghimire, Jennifer M. Fill, Fahong Yu, Raelene M. Crandall, Liu Tie

**Affiliations:** 1 Horticultural Sciences Department, University of Florida, Gainesville, FL 32611, USA; 2 School of Forest, Fisheries, and Geomatics Sciences, University of Florida, Gainesville, FL 32611, USA; jfill@ufl.edu (J.M.F.); raecrandall@ufl.edu (R.M.C.); 3 Bioinformatics, Interdisciplinary Center for Biotechnology Research, University of Florida, Gainesville, FL 32611, USA

**Keywords:** wiregrass, fire, flowering, regrowth

## Abstract

*Aristida beyrichiana* (wiregrass) is a grass species in the southeastern U.S. that exhibits pronounced fire-stimulated flowering, an adaptive trait in fire-prone ecosystems. Although fire-stimulated flowering has been well documented morphologically in this species, its molecular basis remains largely unknown. To investigate species-specific drivers of this adaptation, we performed comparative transcriptomic analyses under natural field conditions, before and approximately seven weeks after a prescribed fire, on wiregrass and two other grass species, *Aristida spiciformis* (bottlebrush threeawn) and *Sporobolus junceus* (pineywoods dropseed), which are not known to rely on fire to induce flowering. All species showed upregulation of conserved stress-related genes after fire, yet wiregrass exhibited a more focused transcriptional response. Notably, genes involved in nitrogen remobilization (*ASN1*, *GDH2*) and carbohydrate metabolism (*SUS4*, *TPS10*, *DPE2*) were uniquely upregulated in wiregrass, suggesting candidate metabolic adaptations that may support rapid post-fire regrowth and flowering. We also detected non-coding RNAs among the differentially expressed genes in wiregrass, pointing to a possible additional layer of regulation in fire-induced responses. Collectively, these results provide new transcriptomic insight into post-fire responses in wiregrass and identify candidate genes and regulatory pathways that require functional validation; because the observed expression differences integrate fire-induced, developmental and seasonal effects, they should not be interpreted as direct fire-specific responses.

## 1. Introduction

Long-lived perennial grasses are a defining component of old-growth savanna understories. In tropical and subtropical locations, C4 grasses both facilitate the spread of fire and recover rapidly through resprouting [1]. These grass-fire dynamics, together with plant spatial patterns and facilitative interactions, influence numerous other plant and animal species and shape ecosystem-level energy and nutrient fluxes [2,3,4,5], thereby affecting the overall savanna structure and function [6]. The demographic persistence of long-lived perennial grasses further contributes to savanna stability across variable fire regimes [7,8,9]. For example, the fire-stimulated flowering of *A. beyrichiana* (*Aristida beyrichiana*) can survive for more than 50 years in unburned pine savannas and resume flowering once fire is reintroduced [10,11]. Similarly, in Australia, *Themeda triandra* plants resprouted following fire after more than a decade without burning [12]. Together, these examples highlight the remarkable resilience of long-lived perennial grasses and their essential role in maintaining the persistence of savanna ecosystems.

Fire-stimulated flowering and seed production are contributors to the long-term demographic persistence of several savanna grasses [9]. This fire-triggered reproductive strategy is widespread across global savannas, including those in Brazil [13], Africa, Australia [14], and North America [15]. Species vary in their degree of dependency on fire for flowering [16]. Some are strongly fire-dependent, flowering primarily or exclusively after fire, whereas others are weakly dependent, producing more flowers after fire yet maintaining low levels of flowering in its absence. A range of cues can induce post-fire flowering, including heat or smoke, changes in light or nutrient availability, and circadian rhythms [14]. In the Southeastern U.S., for example, *A. beyrichiana* produces substantially more flowering culms after late spring and early summer fires than after winter fires [15,17].

Although the evolutionary history of fire-stimulated flowering as an adaptation to fire is well supported [16], the underlying molecular mechanisms remain largely unresolved. Recent studies have shown that karrikins (KARs), smoke-derived butenolides, can stimulate seed germination, alter root architecture, and promote shoot branching in many species following fire [18]. KARs are perceived by the receptor KARRIKIN-INSENSITIVE 2 (KAI2), which functions with the F-box protein MORE AXILLARY GROWTH 2 (MAX2) and the SCF ubiquitin E3-ligase complex to regulate targeted protein degradation through the 26S proteasome [19]. Whether fire-stimulated flowering is also regulated through KAR-mediated signaling remains unclear. However, the broad conservation of KAI2 across plant lineages suggests that this signaling pathway may contribute to multiple aspects of plant adaptation to fire. Importantly, plant responses to fire extend beyond immediate stress signaling and encompass subsequent changes in growth, development, and resource allocation during recovery. In fire-adapted grasses, post-fire regeneration may therefore involve coordinated transcriptional programs associated with vegetative regrowth, developmental transitions, and reproductive responses, including flowering. Consequently, transcriptional differences observed during post-fire recovery may reflect both responses to fire-induced stress and the developmental and physiological states associated with subsequent regrowth. By identifying differentially expressed genes associated with post-fire regrowth and flowering across multiple grass species, this study provides insight into the molecular processes underlying fire-associated adaptations while establishing a foundation for future studies aimed at distinguishing direct fire-responsive signaling from developmental processes associated with post-fire recovery.

Beyond transcriptional control, post-transcriptional and translational regulation provides a rapid means of reprogramming gene expression under stress. Non-coding RNAs (ncRNAs), including microRNAs (miRNAs), long non-coding RNAs (lncRNAs), and small interfering RNAs, can modulate the abundance, stability and translation of target transcripts and have been implicated in plant responses to heat, cold, drought and oxidative stress. At the RNA–protein level, stress granules (cytoplasmic condensates that sequester untranslated mRNAs and RNA-binding proteins) protect transcripts and conserve energy during stress and represent a conserved component of the plant heat-stress response. Because fire exposes plants to intense, transient heat followed by a prolonged recovery phase, such RNA-level mechanisms could contribute to post-fire reprogramming of gene expression; whether ncRNAs or related RNA-level regulators are involved in fire-stimulated flowering has not been examined. We therefore also catalogued the ncRNAs among the differentially expressed genes as a first step toward identifying candidate RNA-level regulators.

In this study, we investigated the molecular mechanisms underlying post-fire regrowth in grass species that differ in their dependence on fire for flowering. *A. beyrichiana*, a C4 grass endemic to pine savannas of the southeastern U.S., exhibits strong fire-stimulated flowering, producing inflorescences almost exclusively after fire, although occasional flowering has been observed in young individuals during their first year [15,20]. To identify candidate genes associated with this response, we compared patterns of gene expression, specifically upregulation or downregulation following fire, in *A. beyrichiana* and two other grass species that do not exhibit strong fire-stimulated flowering: *Aristida spiciformis* (*A. spiciformis*) and *Sporobolus junceus* (*S. junceus*). Our objective was to identify candidate genes and regulatory factors (including non-coding RNAs) potentially involved in fire-stimulated flowering by characterizing and contrasting the transcriptomic response of three savanna grass species before and after fire under natural field conditions.

## 2. Results

### 2.1. Fire-Stimulated Resprouting and Flowering in A. beyrichiana

**Figure 1:**
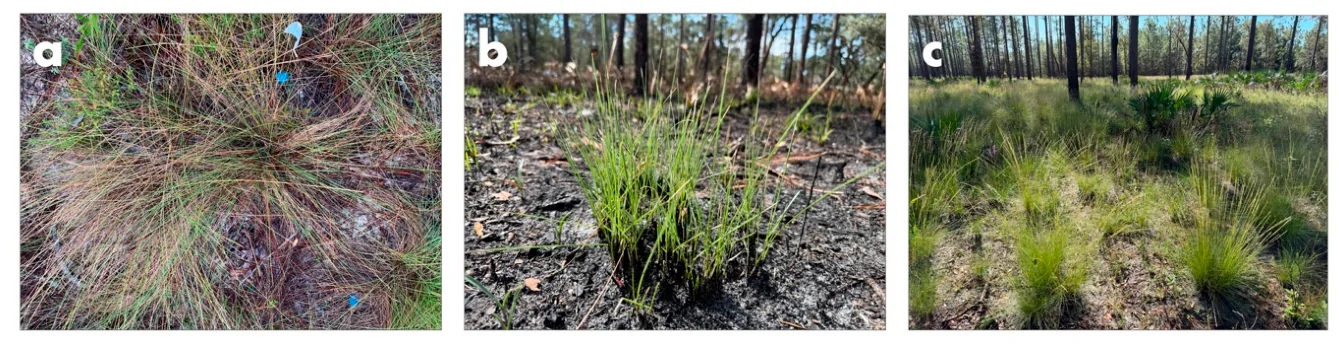
Photographs of *A. beyrichiana* illustrating fire-related phenology and structure. (**a**) Pre-fire plant with no flowering culms and a pronounced layer of dead, wiry leaves that contribute to flammability. (**b**) Regrowth of a wiregrass individual 2 weeks post-fire. (**c**) Our study site in the fall following the fire, showing abundant, flowering wiregrass across the open pine savanna.

*A. beyrichiana* provides an excellent system for studying fire-stimulated growth and reproduction because it reliably resprouts after fire and rapidly transitions into a reproductive phase (Figure 1). Figure 1a shows a pre-fire plant characterized by dense layers of dead, wiry leaves that accumulate as fine fuel and carry fire across pine savannas. Within just two weeks after burning (Figure 1b), wiregrass resprouts vigorously, producing fresh green leaves from basal meristems. By the fall following the fire (Figure 1c), the study site exhibits abundant flowering culms throughout the open pine savanna, highlighting the strong fire-dependent stimulation of reproduction and the predictable post-fire phenological shift in this species. To identify candidate genes and pathways associated with fire-stimulated regrowth and flowering, we compared transcriptomic responses before and after fire in *A. beyrichiana* and two related grass species that do not exhibit strong fire-induced flowering: *A. spiciformis* and *S. junceus*.

### 2.2. Differential Gene Expression in Response to Fire Across Three Savanna Grass Species

**Figure 2:**
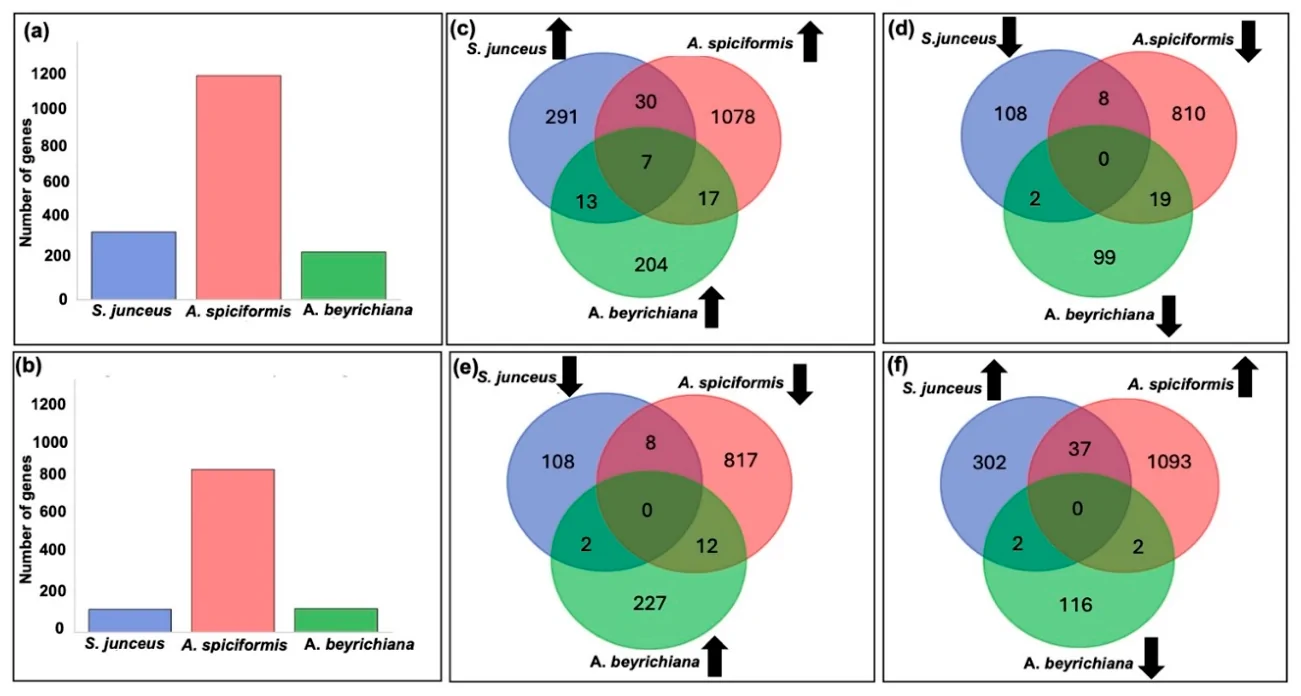
Differential Gene Expression in Response to Fire Across Three Savanna Grass Species. (**a**) Bar plot showing the number of significantly upregulated genes (log_2_FC > 2, FDR < 0.05) in *A. beyrichiana*, *A. spiciformis*, and *S. junceus*. (**b**) Bar plot showing the number of significantly downregulated genes (log_2_FC < −2, FDR < 0.05) across the three species. (**c**) Venn diagram illustrating the overlap of upregulated genes among species. (**d**) Venn diagram showing the overlap of downregulated genes among species. (**e**) Venn diagram showing genes that are oppositely regulated between species (downregulated in *S. junceus* and *A. spiciformis* but upregulated in *A. beyrichiana*). (**f**) Venn diagram showing genes that are oppositely regulated between species (upregulated in *S. junceus* and *A. spiciformis* but downregulated in *A. beyrichiana*).

We quantified species-specific differences in gene regulation across *A. beyrichiana*, *A. spiciformis*, and *S. junceus*. Differential expression was assessed within each species by comparing post-fire samples (collected 48 days after the burn) with pre-fire samples, and genes were then compared across species using the Arabidopsis locus to which their reads were mapped (see Section 5). The DEGs therefore represent the integrated response to fire, regrowth and seasonal progression over this interval, and not fire-specific responses alone (see Section 3). Using thresholds of log_2_FC > 2 for upregulation and log_2_FC < −2 for downregulation, we identified the number of differentially expressed genes in each species (Figure 2a). *A. spiciformis* exhibited the greatest transcriptional response to fire, with 1132 upregulated genes, whereas *A. beyrichiana* and *S. junceus* showed markedly fewer upregulated genes (241 and 341 genes, respectively). This pattern indicates that *A. spiciformis* undergoes a more extensive transcriptional reprogramming following fire, which may contribute to rapid post-fire recovery. A similar trend was observed for downregulated genes: *A. spiciformis* showed the highest number (837), followed by *S. junceus* (118) and *A. beyrichiana* (120) (Figure 2b). These results demonstrate that *A. spiciformis* experiences a broad transcriptional shift, encompassing both gene activation and repression, whereas *A. beyrichiana* and *S. junceus* exhibit more limited responses to fire. Venn diagram analyses further revealed species-specific and shared transcriptional responses among upregulated genes (Figure 2c). *A. spiciformis* possessed the largest set of uniquely upregulated genes (1078), followed by *S. junceus* (291) and *A. beyrichiana* (204). Only seven genes were commonly upregulated across all three species, whereas 17 genes were shared between *A. beyrichiana* and *A. spiciformis*, 19 genes were shared between *A. beyrichiana* and *A. spiciformis*, and 30 genes were shared between *A. spiciformis* and *S. junceus*. The low number of shared upregulated genes suggests that the three species differ largely in their transcriptional responses to fire, noting that genes are matched across species through their Arabidopsis homologs. Downregulated genes also exhibited strong species-specific patterns (Figure 2d). *A. spiciformis* had the greatest number of uniquely downregulated genes (810), followed by *S. junceus* (108) and *A. beyrichiana* (99). Notably, no genes were commonly downregulated across all species. This pronounced divergence in gene repression suggests substantial differences in post-fire stress-response and resource-allocation mechanisms. To identify genes that were downregulated in *S. junceus* and *A. spiciformis* but upregulated in *A. beyrichiana*, we conducted a cross-species comparison of differentially expressed genes (Figure 2e). No genes followed this specific regulatory pattern, indicating that *A. beyrichiana* does not depend on a unique set of transcriptional activations relative to the other two species. However, we detected two genes with opposite regulatory trajectories between *S. junceus* and *A. beyrichiana* (up in one species and down in the other), and 12 genes that showed opposite regulation between *A. spiciformis* and *A. beyrichiana*. These patterns highlight distinct species-specific transcriptional strategies in response to fire, with *A. spiciformis* and *A. beyrichiana* exhibiting the greatest degree of regulatory divergence. Conversely, when examining genes upregulated in both *S. junceus* and *A. spiciformis* but downregulated in *A. beyrichiana* (Figure 2f), we identified two genes that were oppositely regulated between *A. spiciformis* and *A. beyrichiana*, and between *S. junceus* and *A. beyrichiana*. These findings further underscore the heterogeneous molecular responses underlying post-fire recovery across savanna grasses.

### 2.3. ncRNAs and Protein-Coding Genes in Wiregrass Post-Fire Response

**Figure 3:**
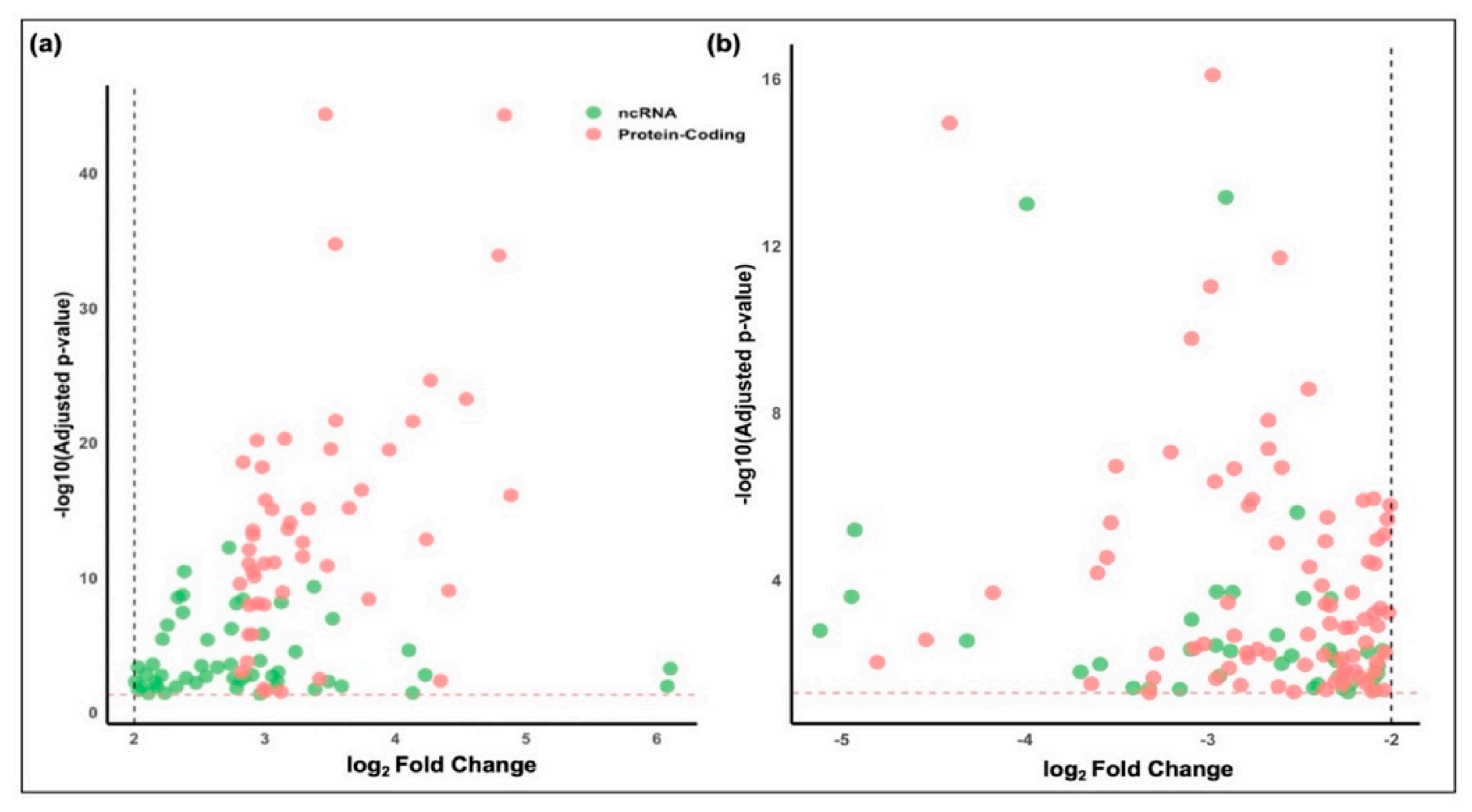
Differential expression of ncRNAs and protein-coding genes in the post-fire response of *A. beyrichiana*. (**a**). Volcano plot of upregulated genes (log_2_FC > 2), highlighting protein-coding genes (red) and ncRNAs (blue). (**b**) Volcano plot of downregulated genes (log_2_FC < −2), illustrating a similar pattern, with many protein-coding genes exhibiting strong repression and a substantial subset of ncRNAs also showing reduced expression. The red horizontal dashed line represents the significance threshold (log10 adjusted *p*-value = 0.05). The black vertical dashed lines indicate the fold-change thresholds used to define biologically meaningful differential expression (log_2_FC = ±2).

**Figure 4:**
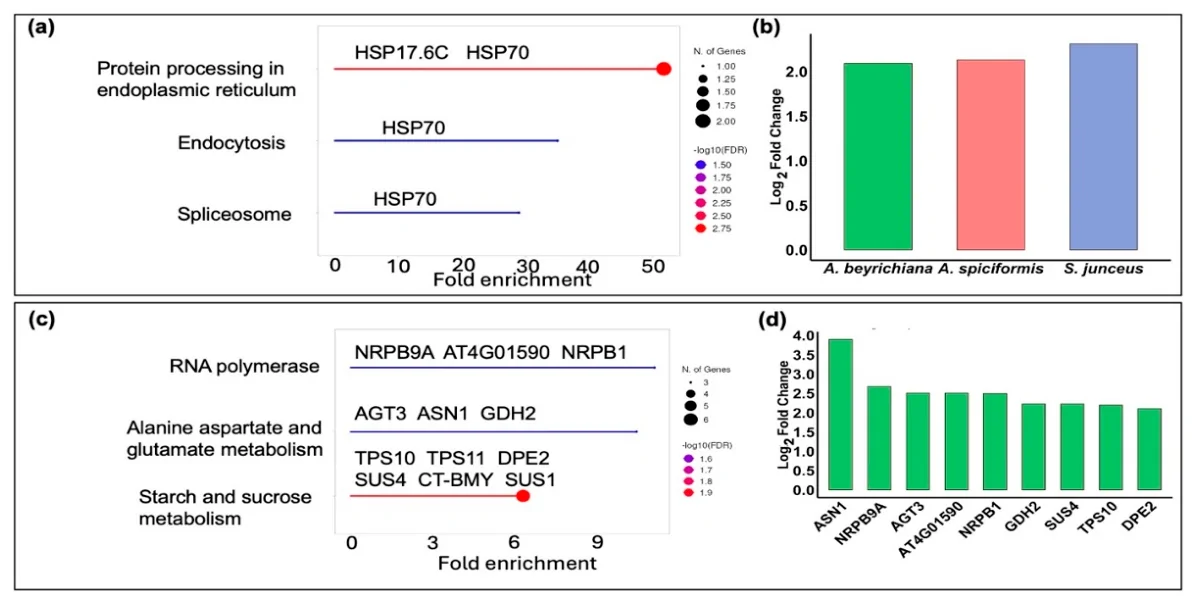
Functional enrichment and species-specific gene expression patterns following fire. (**a**). Gene Ontology (GO) terms enriched among genes commonly upregulated across all three species. (**b**). Fold-change expression of *HSP70* across species. (**c**). GO term enrichment for genes uniquely upregulated in *A. beyrichiana*. (**d**). Expression patterns of selected genes uniquely upregulated in *A. beyrichiana*.

Among the differentially expressed genes (DEGs), we identified a significant number of non-coding RNAs (ncRNAs), including long non-coding RNAs (lncRNAs), microRNAs (miRNAs), and transfer RNAs (tRNAs). Given their potential regulatory functions, we examined ncRNA expression patterns alongside those of protein-coding genes to assess their possible involvement in the post-fire response of *A. beyrichiana*. Analysis of upregulated DEGs (log_2_FC > 2) revealed increased expression of both protein-coding genes and ncRNAs (Figure 3a). Several strongly upregulated ncRNAs, such as lncRNAs and miRNAs, including the microRNA precursor *MIR169C* (*AT5G39635*; see below), may participate in gene regulation, by modulating either transcriptional or post-transcriptional regulation, potentially modulating stress-response and growth pathways following a fire. The upregulation of multiple tRNAs may reflect elevated translational demand, possibly supporting the synthesis of proteins required for post-fire recovery and the initiation of flowering; however, because our libraries were prepared from poly(A)-enriched RNA, non-polyadenylated ncRNAs such as tRNAs and mature miRNAs are expected to be under-represented, and their quantification should be regarded as tentative (see Section 3). Upregulated protein-coding genes included those encoding the molecular chaperones HSP70 and HSP17.6C (upregulated in all three species), the nitrogen-metabolism genes *ASN1* and *GDH2*, and the carbohydrate-metabolism genes *SUS4*, *TPS10*, and *DPE2* (as described in the following section; Figure 4), which is consistent with, but does not demonstrate, activation of proteostasis and nitrogen- and carbon-remobilization pathways during regrowth. Conversely, evaluation of downregulated DEGs (log_2_FC < −2) showed coordinated repression of both ncRNAs and protein-coding genes (Figure 3b). The downregulation of specific ncRNAs may reflect a reorganization of transcriptional control, enabling the selective activation of adaptive pathways under post-fire conditions. Collectively, these findings suggest that ncRNAs may contribute to the transcriptional landscape of *A. beyrichiana* during post-fire recovery. The coordinated up- and downregulation of ncRNAs alongside protein-coding genes is consistent with a possible regulatory role for non-coding elements in regrowth and flowering following fire; this hypothesis remains to be tested functionally.

### 2.4. Conserved and Unique Genes Upregulated in the Post-Fire Response of A. beyrichiana

Among the differentially expressed genes (DEGs) identified across species, only seven genes were commonly upregulated in *A. beyrichiana*, *A. spiciformis*, and *S. junceus* (Figure 4a). Gene Ontology (GO) enrichment analysis of these shared DEGs indicated involvement in essential cellular processes, including protein processing in the endoplasmic reticulum, endocytosis, and spliceosome function. Notably, the molecular chaperones *HSP70* and *HSP17.6C* were highly enriched, suggesting a conserved role in protein folding and stress adaptation following fire across all three species. To further assess the importance of *HSP70*, we examined its expression patterns across species (Figure 4b). *HSP70* was significantly upregulated in all three species, consistent with its role in mitigating stress and maintaining cellular homeostasis; however, because post-fire samples were collected in mid-summer, induction of heat-shock genes may reflect fire-associated heat, other post-fire or seasonal stresses, or a combination of these (see Section 3). The consistent upregulation of heat shock proteins is consistent with a shared, possibly evolutionarily conserved, mechanism supporting post-fire recovery in savanna grasses. We further examined the 204 uniquely upregulated genes in *A. beyrichiana* (Figure 4c). GO analysis indicated enrichment in pathways related to RNA polymerase activity, amino acid metabolism (including alanine, aspartate, and glutamate), and starch and sucrose metabolism, suggesting that *A. beyrichiana* may employ a species-specific transcriptional program that facilitates metabolic adaptation, post-fire regrowth, and flowering. Notably, key genes associated with these metabolic pathways, such as *ASN1*, *NRPB9A*, and *AGT3*, exhibited elevated transcript levels following fire (Figure 4d). The increased expression of *ASN1* and *GDH2* points to a potential adaptation for optimizing nitrogen utilization. In contrast, upregulation of starch and sucrose metabolism genes (*SUS4*, *TPS10*, and *DPE2*) is consistent with possible carbohydrate mobilization to support regrowth; we note, however, that transcript abundance alone does not demonstrate changes in enzyme activity, metabolite pools or metabolic flux, which were not measured here. Collectively, these results are consistent with both conserved and species-specific transcriptional responses to fire, and identify heat shock proteins, nitrogen- and carbohydrate-metabolism genes, and transcriptional regulators as candidates for roles in post-fire recovery among these species.

### 2.5. Oppositely Regulated Genes Across A. beyrichiana, A. spiciformis, and S. junceus

**Figure 5:**
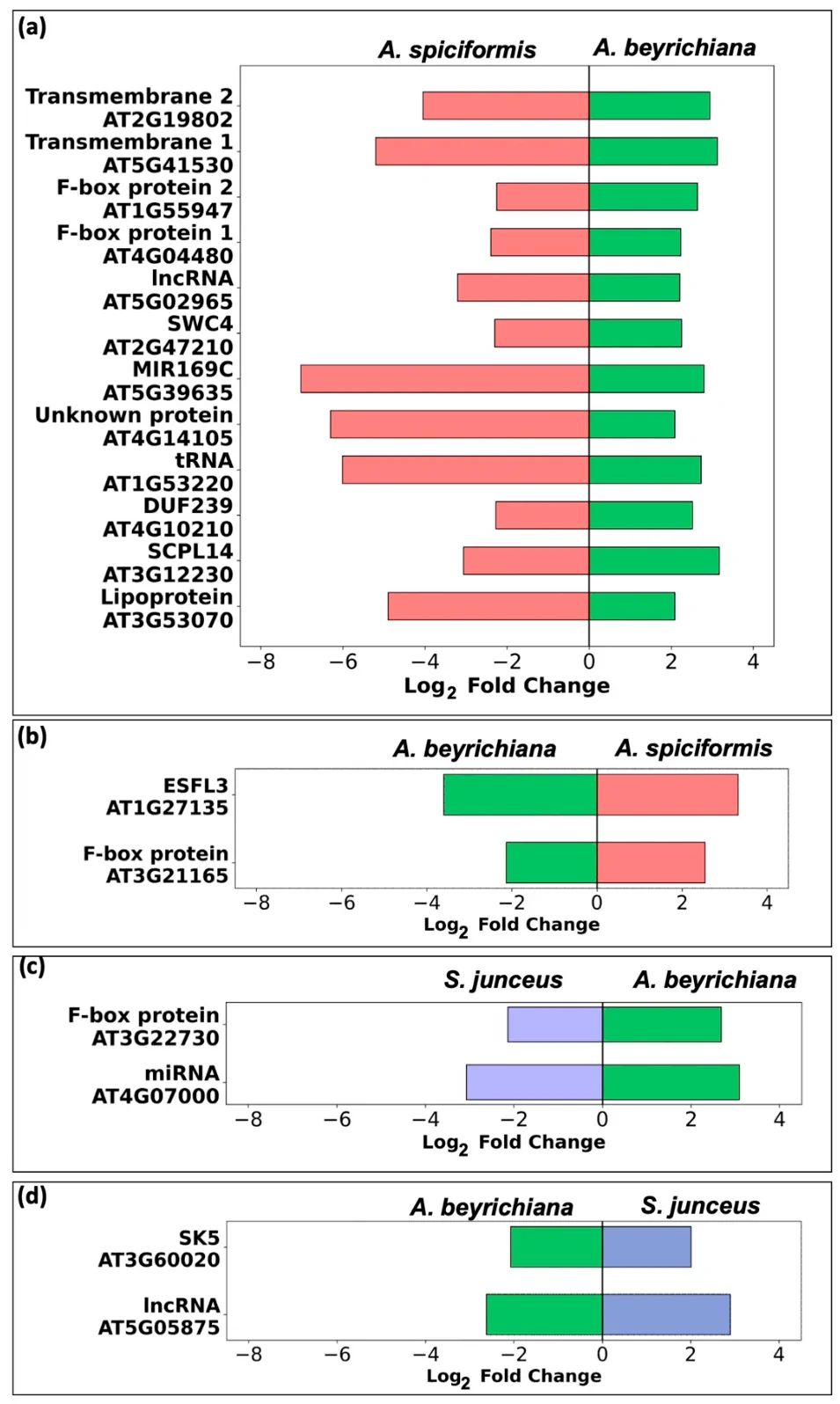
Oppositely regulated genes across *A. beyrichiana*, *A. spiciformis*, and *S. junceus* (**a**) Genes exhibiting opposite regulation between *A. beyrichiana* and *A. spiciformis*, with upregulated genes in *A. beyrichiana* shown in blue and corresponding downregulated genes in *A. spiciformis* shown in red. (**b**) Additional genes showing inverse expression patterns between *A. beyrichiana* and *A. spiciformis*. (**c**) Genes exhibiting opposite regulation between *A. beyrichiana* and *S. junceus*. (**d**) Additional genes showing inverse expression patterns between *A. beyrichiana* and *S. junceus*.

Fire-induced regrowth in *A. beyrichiana*, *A. spiciformis*, and *S. junceus* is accompanied by distinct, species-specific transcriptional changes. To further resolve these differences, we identified genes exhibiting opposite regulatory patterns among species (Figure 5a–d). Several genes displayed inverse expression between *A. beyrichiana* and *A. spiciformis*, with transcripts upregulated in one species but downregulated in the other (Figure 5a). These genes encompass diverse functional categories, including non-coding RNAs (lncRNA, tRNA, miRNA), transport proteins (transmembrane proteins), and metabolic regulators (F-box proteins, lipoproteins). Notably, *AT5G39635* (*MIR169C*) and *AT4G14105* (unknown protein) were strongly upregulated in *A. beyrichiana* but significantly downregulated in *A. spiciformis*, suggesting divergent post-fire recovery strategies. Further analysis of these inversely regulated genes confirmed significant transcriptional divergence between the two species (Figure 5b). Genes such as *ESFL3* (*AT1G27135*) and F-box protein (*AT3G21165*) were positively regulated in *A.* spiciformis but downregulated in *A. beyrichiana*, indicating species-specific roles in stress adaptation and metabolic regulation. We also identified genes exhibiting opposite regulation between *A. beyrichiana* and *S. junceus* (Figure 5c). For example, *AT5G05875* (lncRNA) and *AT3G60020* (SK5) were upregulated in *S. junceus* but downregulated in *A. beyrichiana*, suggesting that *S. junceus* employs distinct molecular strategies for post-fire recovery. A second set of oppositely regulated genes (Figure 5d) further supports this pattern, with SK5 again showing upregulation in *S. junceus* but downregulation in *A. beyrichiana*, reinforcing the conclusion that transcriptional responses to fire diverge substantially across these species. qRT-PCR analysis supported the RNA-seq-based expression patterns of *ESFL3* and *SCPL14* in *A. spiciformis* and *A. beyrichiana* (Supplementary Figure S1a–d). In *A. spiciformis*, *ESFL3* expression increased following fire, whereas *SCPL14* expression decreased. In contrast, *A. beyrichiana* showed a slight reduction in *ESFL3* expression, and a modest increase in *SCPL14* expression. These opposing expression patterns closely paralleled the RNA-seq results, providing independent validation for the transcriptomic data and reinforcing the observed species-specific regulatory responses to fire.

### 2.6. Functional Enrichment of DEGs in S. junceus and A. spiciformis

To further characterize species-specific molecular responses to fire-stimulated regrowth, we conducted functional enrichment analyses on genes exclusively upregulated or downregulated in *S. junceus* and *A. spiciformis*. In *S. junceus*, genes uniquely upregulated following fire showed strong enrichment in galactose metabolism, indicating a pronounced metabolic shift towards carbohydrate utilization (Supplementary Figure S2a). This pattern suggests that *S. junceus* may rely on galactose-derived energy production and associated cellular repair processes during post-fire recovery. In contrast, genes exclusively downregulated in *S. junceus* were enriched in pathways related to photosynthesis-antenna proteins, sphingolipid metabolism, cysteine and methionine metabolism, and amino sugar metabolism (Supplementary Figure S2b). Downregulation of photosynthetic genes may reflect a temporary suppression of light-dependent reactions under post-fire stress, while reduced activity in amino sugar and sphingolipid metabolic pathways could indicate shifts in cell-wall remodeling and membrane stabilization during recovery. Genes exclusively upregulated in *A. spiciformis* were strongly enriched for oxidoreductase activity, electron transfer activity, and NADH dehydrogenase activity, particularly through proton transmembrane transporter activity (Supplementary Figure S2c). These enrichments suggest that *A. spiciformis* prioritizes mitochondrial respiration and redox regulation as central components of its fire-stimulated regrowth. The observed enrichment in NADH dehydrogenase activity and proton transmembrane transporters likely reflects increased energetic demands, supporting rapid regrowth through enhanced ATP production.

## 3. Discussion

Across species, the identification of genes exhibiting opposite regulation points to a distinct post-fire transcriptional response of *A. beyrichiana* relative to *A. spiciformis* and *S. junceus*. Species-specific enrichment of metabolic and regulatory pathways is consistent with diverse strategies during post-fire recovery. Taken together, these results are consistent with distinct metabolic reprogramming among the three species, with *S. junceus* showing transcriptional signatures of enhanced carbohydrate metabolism, and *A. spiciformis* showing upregulation of mitochondrial energy pathways and oxidative stress responses.

**Figure 6:**
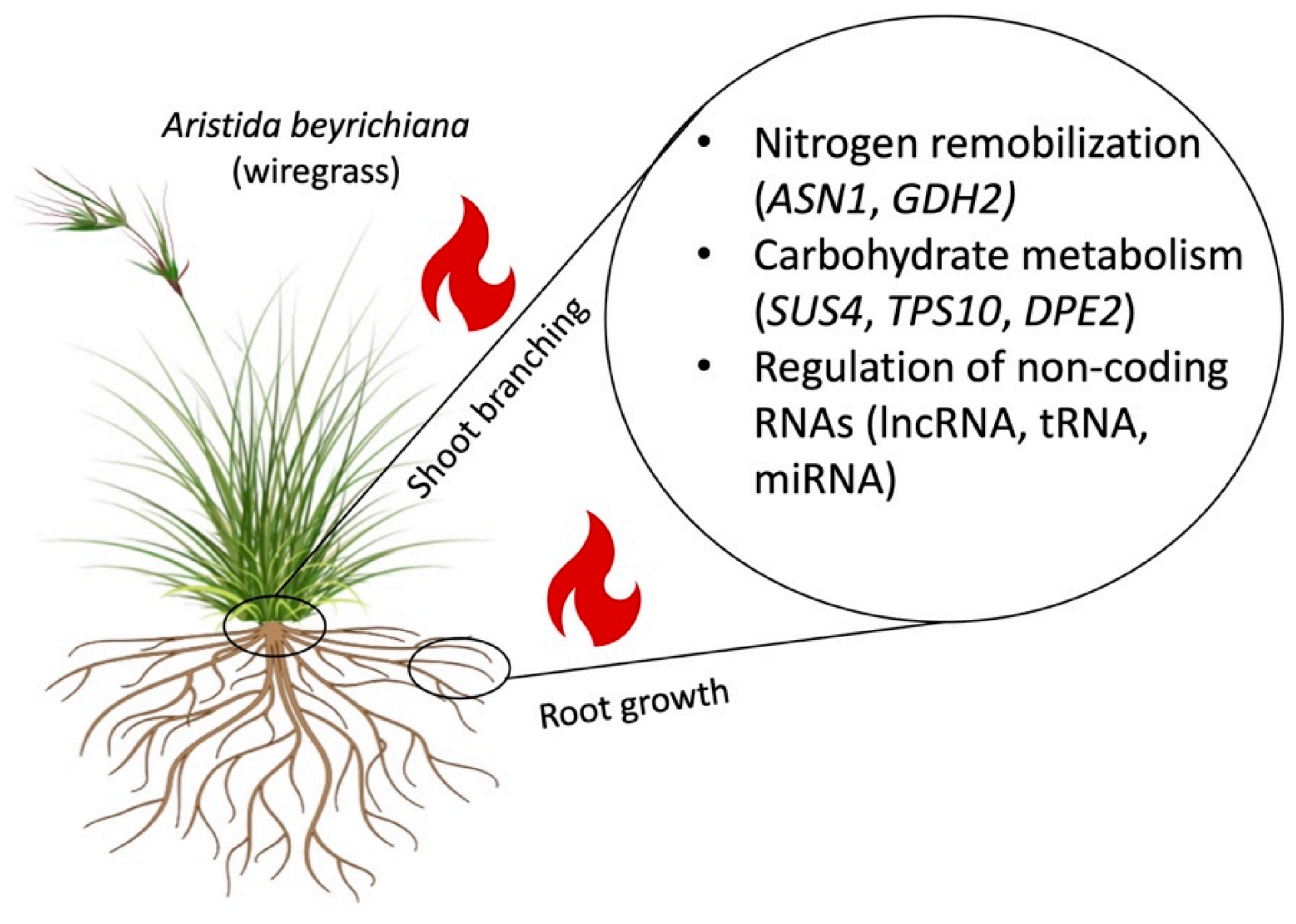
Schematic summary of fire-stimulated pathways in wiregrass.

These contrasting responses point to divergent survival strategies and physiological trade-offs among fire-adapted grasses. Our RNA-seq analysis revealed substantial transcriptional variation among the three grass species following fire exposure. *A. spiciformis* exhibited the most extensive transcriptional reprogramming, with more than 1100 genes significantly upregulated, in contrast to the more moderate responses observed in *A. beyrichiana* and *S. junceus*. These differences may reflect ecological or morphological variation between species. One of the most prominent conserved responses across all three species was the induction of heat shock proteins (HSPs), particularly *HSP70* and *HSP17.6C*. HSPs serve as molecular chaperones, promoting protein folding, stabilization, and degradation under stress conditions [21]. In addition to their established role in thermal tolerance, *HSP70*s are implicated in plant immunity, where their activity is regulated by phytohormones such as cytokinins and various post-translational modifications [22]. The strong induction of HSPs across species underscores their central role in maintaining proteostasis and promoting physiological resilience in response to fire-induced stress. In *A. beyrichiana*, the significant upregulation of *ASN1* and *GDH2* suggests enhanced nitrogen remobilization, a process critical for efficient nutrient recycling and post-fire regrowth. A comparable mechanism has been documented in wheat, where the NAC transcription factor *NAM-B1* activates nitrogen metabolism genes, including *ASN1* and *GDH*, to support nitrogen recycling during senescence [23]. This parallel implies that *A. beyrichiana* may employ similar regulatory pathways to optimize nitrogen use during post-fire regrowth. Additionally, increased expression of carbohydrate metabolism genes, such as *SUS4*, *TPS10*, and *DPE2*, indicates a metabolic strategy focused on the rapid mobilization of stored carbon reserves. In *Arabidopsis thaliana*, the trehalose 6-phosphate signaling pathway, mediated by *TPS1*, integrates carbon and nitrogen signals to regulate flowering via *FLOWERING LOCUS C* (*FLC*) [24], suggesting that *A. beyrichiana* may rely on analogous metabolic coordination to support post-fire regrowth and reproductive transitions. Our analysis also revealed a substantial number of non-coding RNAs (ncRNAs), including long non-coding RNAs (lncRNAs), microRNAs (miRNAs), and transfer RNAs (tRNAs), among the differentially expressed genes. The upregulation of lncRNAs and miRNAs in *A. beyrichiana* points to their involvement in stress signaling and post-transcriptional regulation. Recent studies have highlighted the importance of diverse ncRNAs, including siRNAs, circRNAs, and lncRNAs, in orchestrating plant responses to heat, cold, and oxidative stress [25]. The presence of similar ncRNAs in *A. beyrichiana* after fire suggests a conserved regulatory role in enhancing transcriptomic flexibility and environmental resilience (Figure 6).

Caveats and interpretation. Several features of the study design constrain the inferences that can be drawn from these data. First, post-fire samples were collected 48 days (about seven weeks) after the burn, whereas pre-fire samples were collected 12 days before and immediately before the burn, and no unburned control plants were sampled concurrently; fire-specific effects therefore cannot be separated from regrowth, developmental progression, and seasonal change between June and August in temperature, photoperiod and rainfall, any of which could alter gene expression independently of fire. Second, plants were sampled under natural field conditions, where uncontrolled biotic and abiotic factors (e.g., heat and drought episodes, herbivory, pathogens, and post-fire changes in light and nutrient availability) can induce stress-related genes; the induction of heat-shock genes, in particular, may reflect fire-associated heat, seasonal heat, or other stresses. Third, whole plants were pooled for RNA extraction, so tissue- or organ-specific responses (e.g., of shoot meristems or inflorescence primordia) cannot be resolved. Fourth, reads from all three species were mapped to the *Arabidopsis thaliana* genome, which is distantly related to grasses; this captures conserved genes with recognizable Arabidopsis homologs but not lineage-specific genes. It may merge reads from grass paralogs onto a single Arabidopsis locus, and treat genes as equivalents across species on the basis of shared Arabidopsis homology rather than inferred orthology. Fifth, libraries were prepared from poly(A)-enriched RNA, so non-polyadenylated ncRNAs are under-represented and the ncRNA findings should be regarded as preliminary. Finally, our inferences about nitrogen and carbohydrate metabolism rely on transcript abundance alone; we did not measure enzyme activities, metabolites or fluxes. The proposed roles of these pathways and of ncRNAs should therefore be regarded as hypotheses requiring functional validation.

These findings also have broader ecological relevance. *A. beyrichiana* and other dominant grasses can strongly influence fire regimes and ecosystem dynamics in pine savannas [4,9,26]. Future research should focus on the functional validation of candidate genes and ncRNAs identified here, particularly those exhibiting contrasting regulation among species. Experimental manipulation of these molecular pathways, coupled with phenotypic assessment under controlled burn scenarios, will be essential for elucidating genetic architecture underlying fire-stimulated regrowth and reproduction in fire-adapted grasses such as *A. beyrichiana*. Complementary approaches, such as a de novo transcriptome or genome assembly for wiregrass to allow orthology-based cross-species comparison (or mapping to genomes of more closely related grasses), total-RNA and small-RNA sequencing to profile ncRNAs, and sampling schemes that include concurrently collected unburned controls and multiple post-fire time points, would help separate fire-specific from developmental and seasonal responses. This study provides the first transcriptomic view of candidate molecular processes underlying fire-stimulated flowering in *A. beyrichiana*, a species strongly dependent on fire as a reproductive cue. It also describes post-fire transcriptional responses in two additional savanna grasses: *A. spiciformis* and *S. junceus*. Our findings suggest that the coordinated upregulation of genes involved in nitrogen remobilization, carbohydrate metabolism, and the regulation of non-coding RNAs may contribute to fire-triggered flowering in *A. beyrichiana*; these candidate pathways should be treated as hypotheses for future functional tests. Together, these pathways could facilitate the allocation of energy and the developmental shifts required for reproduction after fire disturbance. As a dominant component of pine savanna groundcover, *A. beyrichiana* plays a crucial role in post-fire ecosystem recovery; thus, understanding its fire-responsive flowering mechanisms provides valuable insights for managing and conserving fire-adapted plant communities.

## 4. Conclusions

An important consideration in interpreting these transcriptional responses is that post-fire samples were collected approximately seven weeks after the prescribed burn, when plants were actively undergoing regrowth. Thus, the observed transcriptional differences should not be interpreted exclusively as direct molecular responses to fire. They likely integrate multiple biological processes associated with post-fire recovery, including stress responses, tissue regeneration, resource allocation, developmental progression, and differences in physiological condition. In particular, species that differ in their capacity for post-fire regrowth or flowering may also differ in developmental state at the time of sampling, potentially contributing to the observed differences in gene expression. Because plant size and developmental stage were not experimentally matched between pre- and post-fire samples, the present study cannot fully separate transcriptional responses directly induced by fire from those associated with subsequent growth and developmental transitions. Rather, our results characterize the integrated transcriptional state associated with post-fire recovery under natural field conditions. This distinction is particularly relevant for interpreting genes associated with growth, development, and reproduction, as their differential expression may represent components of the post-fire regeneration strategy rather than direct responses to fire itself. Future studies incorporating multiple sampling points immediately after fire and throughout regrowth, together with measurements of plant size, developmental stage, and physiological status, will be necessary to distinguish acute fire responses from developmental changes associated with recovery.

## 5. Materials and Methods

### 5.1. Plant Materials

We collected grasses for this study from a xeric pine savanna at the University of Florida’s Ordway-Swisher Biological Station in Putnam County, Florida (29°41′ N, 82°00′ W). The region has a humid, warm temperate climate, with a mean annual temperature of 20 °C and approximately 1432 mm of annual precipitation [27]. Soils at the site are primarily Entisols. Longleaf pine (*Pinus palustris*) dominates the overstory, while bunchgrasses such as *A. beyrichiana*, *S. junceus*, and *Andropogon* spp. characterize the understory. The study area has been managed with growing-season prescribed fires (May–July) at 2–3-year intervals for several decades. During the study year, the site was burned on 27 June 2023. For each species and sampling time point, three independent plants were collected as biological replicates. To standardize tissue collection across species and sampling periods, entire individual plants, including both above- and belowground tissues, were harvested. Soil was carefully washed from the roots, and the whole plant was used for RNA extraction. Thus, transcriptomic profiles represent whole-plant responses to fire rather than responses of individual tissues or organs. The same whole-plant sampling and processing procedure was applied consistently across species and sampling periods. Pre-fire samples were collected on 15 June 2023 (12 days before the burn) and immediately before the prescribed burn on 27 June 2023, whereas post-fire samples were collected on 14 August 2023 (48 days after the burn) to characterize early regrowth responses. Samples were collected within a consistent time window during the day and from individuals growing within the same study area to reduce variation associated with diel and local environmental conditions. Samples collected on the two pre-fire dates were analyzed separately to assess temporal variation in gene expression prior to the fire and to establish the pre-fire baseline for comparison with post-fire responses. Following collection and processing, whole-plant tissues were immediately stored at −80 °C until RNA extraction.

### 5.2. RNA Extraction, Library Preparation, and Sequencing

Total RNA was isolated using the TRIzol reagent (Invitrogen, Carlsbad, CA, USA) following a modified protocol. RNA integrity was assessed by agarose gel electrophoresis, and concentration was quantified using a spectrophotometer. RNA integrity numbers (RINs) were obtained using an Agilent 4150 TapeStation system (Agilent, Santa Clara, CA, USA), and samples were further purified using a commercial RNA cleanup kit when necessary. To remove residual genomic DNA contamination, 18 µL of total RNA was treated with 2 µL of rDNAse (Invitrogen, Carlsbad, CA, USA) and incubated at room temperature for 15–30 min prior to downstream applications. RNA sequencing libraries were prepared by the ICBR Gene Expression & Genotyping Core Lab using the NEBNext^®^ Ultra™ Directional RNA Library Prep Kit for Illumina (New England Biolabs, Ipswich, MA, USA) following the manufacturer’s instructions. For each sample, approximately 1000 ng of high-quality total RNA (RIN ≥ 6) was used for library construction. mRNA was enriched with the NEBNext Poly(A) mRNA Magnetic Isolation Module (E7490), after which libraries were generated using the NEBNext Ultra II Directional Library Prep Kit (E7760). RNA fragmentation was carried out by incubating samples at 94 °C for 5 min in the NEBNext First Strand Synthesis Buffer. First-strand cDNA synthesis was performed using reverse transcriptase with oligo (dT) primers, followed by second-strand synthesis to generate double-stranded cDNA. Libraries then underwent end-repair, adaptor ligation, and PCR enrichment (8 cycles). Amplified, uniquely barcoded libraries were purified with Agencourt AMPure beads (Beckman Coulter, Brea, CA, USA. A63881). Final pooled libraries were sequenced on an Illumina NovaSeq 6000 platform (Illumina Inc., San Diego, CA, USA) using 150-cycle paired-end sequencing.

### 5.3. RNA Data Analysis

Because no publicly available genome exists for *A. beyrichiana*, RNA-seq reads were aligned to the *Arabidopsis thaliana* reference genome. *Arabidopsis* was selected due to its comprehensive genome annotation and the conservation of molecular pathways involved in plant stress responses and growth. Raw sequencing reads generated on the Illumina NovaSeq 6000 platform were trimmed for adaptors and low-quality bases (Phred score < 20) using Cutadapt software [28] and reads shorter than 60 bases after trimming were removed from further analysis. High-quality reads were mapped to both the rice (*O. sativa* cv. Nipponbare genome, IRGSP 1.0, https://panoryza.org/node/3, accessed on 21 December 2023) and *Arabidopsis thaliana* reference genomes using STAR software (v2.7.9a) [29]. However, alignment to the rice genome resulted in a substantially lower read mapping rate compared with Arabidopsis. Because reference genomes and annotations were unavailable for the three grass species, RNA-seq reads were mapped to the well-annotated *Arabidopsis thaliana* reference genome to identify conserved genes and pathways associated with plant growth and stress responses. This is likely due to the better gene annotation available in Arabidopsis for the pathways examined in this study. Because our downstream analyses relied on accurate transcript quantification and functional interpretation, particularly functional annotation and GO/KEGG enrichment, the higher mapping efficiency and more comprehensive functional annotation in Arabidopsis provided more reliable results. Therefore, we used Arabidopsis as the reference genome for subsequent analyses. We acknowledge that Arabidopsis and the grasses diverged more than 100 million years ago; mapping to such a distant reference primarily captures conserved genes with recognizable Arabidopsis homologs, whereas lineage-specific or highly divergent genes are not represented. Because differential expression was calculated within each species (post-fire versus pre-fire) using the same reference and mapping parameters, systematic mapping biases that are constant within a species are expected to partly cancel out; the main impact is on which genes are detectable and on comparisons of gene numbers among species. Genes were compared across species using the Arabidopsis locus identifier (TAIR10) to which reads from each species were assigned; that is, genes were treated as shared between species when they mapped to the same Arabidopsis locus. We did not infer orthology by phylogenetic or reciprocal-best-hit methods, and reads from multiple grass paralogs may map to a single Arabidopsis locus. “Shared” and “oppositely regulated” genes therefore refer to genes with a common best-matching Arabidopsis homolog rather than to strictly defined orthologs. Mapping results were processed with HTSeq (v0.11.2) [30], Samtools, and custom ICBR scripts to remove PCR duplicates and quantify uniquely mapped reads. FDR was calculated based on three biological replicates. Principal Component Analysis (PCA) was conducted using the R package (v4.1.3) to detect outlier samples based on gene expression profiles. Cross-species mapping was performed using STAR with relaxed alignment stringency (--outFilterMismatchNmax 30, --outFilterMismatchNoverLmax 0.3, --outFilterMultimapsNmax 100, --seedSearchStartLmax 18, --seedSearchStartLmaxOverLread 0.18, and --scoreMinOverLread 0.4). Gene expression was quantified independently for each species, and downstream analyses focused on differentially expressed genes with an FDR < 0.05. Functional interpretation of DEGs was conducted using ShinyGO (v0.741) [31] to assess Gene Ontology (GO) term enrichment and KEGG pathway involvement, allowing the identification of major biological processes and molecular pathways affected by fire.

This is likely due to the better gene annotation available in Arabidopsis for the pathways examined in this study. Because our downstream analyses relied on accurate transcript quantification and functional interpretation, particularly for the characterization of selected mutants, the higher mapping efficiency and more comprehensive functional annotation in Arabidopsis provided more reliable results. Therefore, we used Arabidopsis as the reference genome for subsequent analyses. Mapping results were processed with HTSeq (v0.11.2) [30], Samtools, and custom ICBR scripts to remove PCR duplicates and quantify uniquely mapped reads. FDR was calculated based on three biological replicates. Principal Component Analysis (PCA) was conducted using the R package (v4.1.3) to detect outlier samples based on gene expression profiles. Differentially expressed genes (DEGs) were then identified by applying log2 fold-change and statistical significance thresholds. Functional interpretation of DEGs was conducted using ShinyGO (v0.741) [31] to assess Gene Ontology (GO) term enrichment and KEGG pathway involvement, allowing the identification of major biological processes and molecular pathways affected by fire.

### 5.4. Quantitative PCR (qPCR) Validation

Quantitative PCR (qPCR) was conducted to validate the expression patterns of two genes identified by RNA-seq as oppositely regulated between *A. spiciformis* and *A. beyrichiana*: *ESFL3* (*AT1G27135*) and *SCPL14* (*AT3G12230*). First-strand cDNA was synthesized from DNase-treated total RNA using SuperScript™ IV Reverse Transcriptase (Thermo Fisher Scientific, Waltham, MA, USA) according to the manufacturer’s instructions. Each biological replicate (*n* = 3) was analyzed with three technical replicates to ensure measurement precision. Actin2 was used as the reference gene for normalization. qPCR reactions were performed in 20 µL containing the cDNA amount and primer concentration using the PowerUp SYBR Green Master Mix (Thermo-Fisher, Waltham, MA, USA) on Bio-Rad Real-Time detection systems. Relative gene expression levels were calculated using the ΔΔCT method [32], comparing post-fire with pre-fire samples within each species.

## Data Availability

The dataset has been successfully submitted to the NCBI Gene Expression Omnibus (GEO) under accession number GSE317189 (tracking ID #25543592).

## Supplementary Materials

The following supporting information can be downloaded at https://www.mdpi.com/article/10.3390/ijms27219590/s1.
