# Supplementary material for: Transcriptional Responses to Fire in Three Savanna Grass Species: Candidate Pathways for Fire-Stimulated Flowering
Source: Int J Mol Sci. 2026 Oct 27;27(21):9590. doi: 10.3390/ijms27219590 (PMC13763419; doi:10.3390/ijms27219590)
Supplement: Supplementary file 1 [file ijms-27-09590-s001.zip › ijms-4594700-supplementary.pdf]

Supplementary Materials

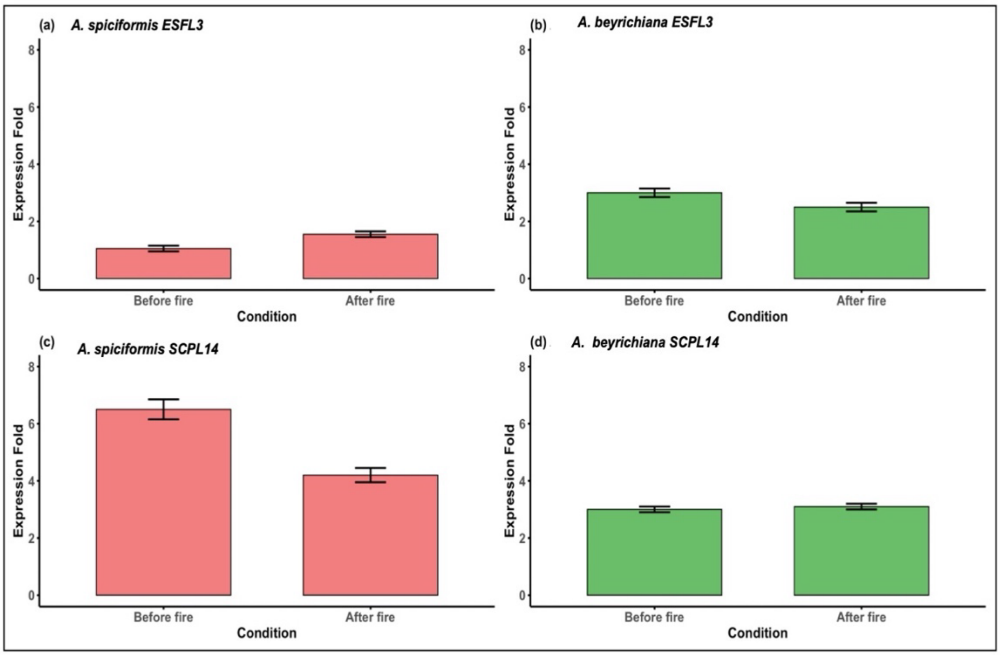

Figure S1. qRT-PCR validation of oppositely regulated genes *A. spiciformis* and *A. beyrichiana* before and after fire. Bars represent mean expression fold-change in expression (normalized to actin) ± standard error (SE). (**a**) ESFL3 expression significantly increased *A. spiciformis* following fire. (**b**), ESFL3 expression decreased in *A. beyrichiana* following fire. (**c**) SCPL14 expression decreased in *A. spiciformis* following fire. (**d**) SCPL14 expression increased in *A. beyrichiana* following fire.

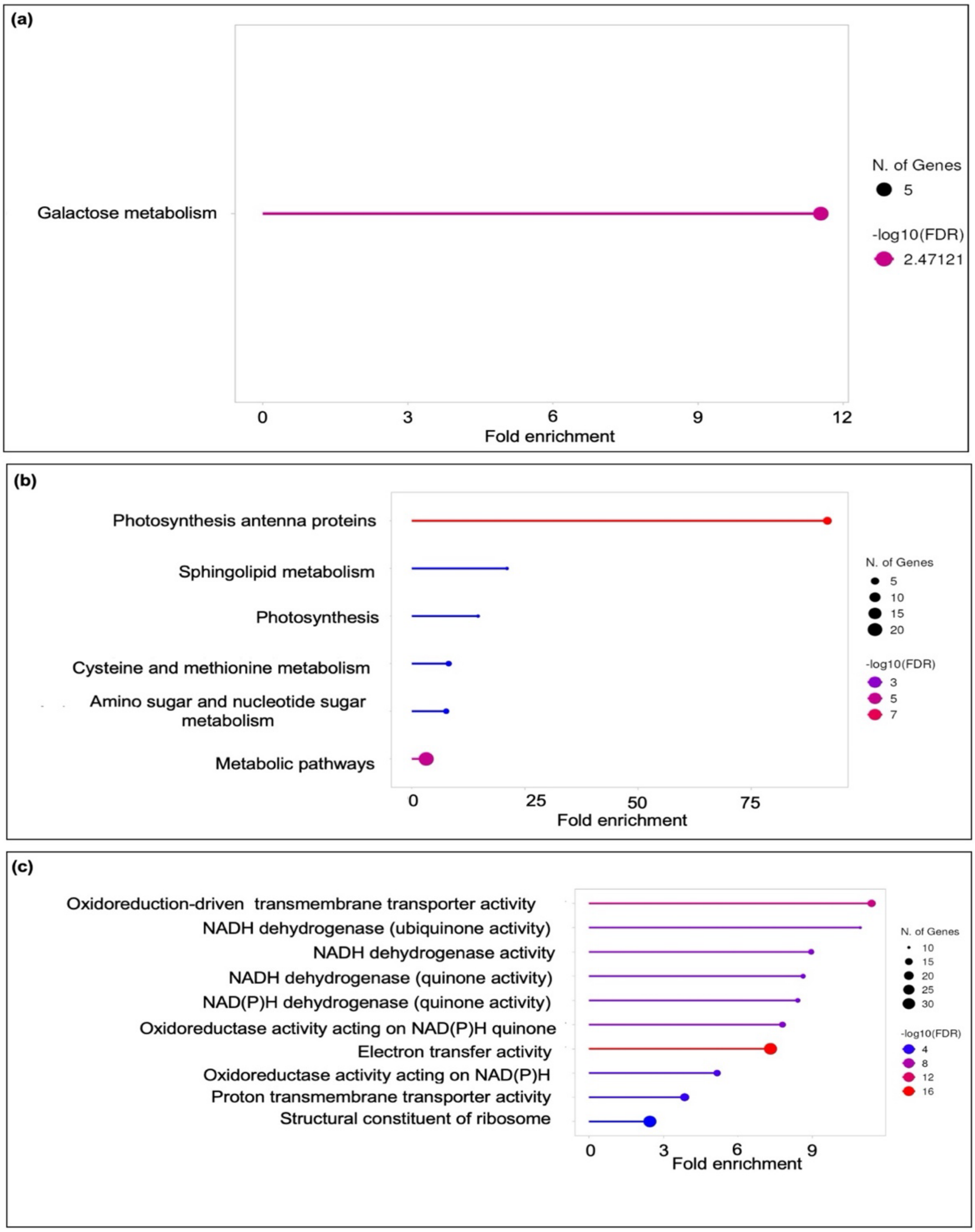

Figure S2: Functional enrichment of differentially expressed genes in *S. junceus* and *A. spiciformis*. (**a**) Enriched pathways among genes uniquely upregulated in *S. junceus* following fire. (**b**) Enriched pathways among genes uniquely downregulated in *S. junceus*. (**c**) Enriched molecular functions among genes uniquely upregulated in *A. spiciformis*.
